# Plasma chemokines are biomarkers of disease severity, higher bacterial burden and delayed sputum culture conversion in pulmonary tuberculosis

**DOI:** 10.1038/s41598-019-54803-w

**Published:** 2019-12-03

**Authors:** Nathella P. Kumar, Kadar Moideen, Arul Nancy, Vijay Viswanathan, Basavaradhya S. Shruthi, Shanmugam Sivakumar, Mohan Natarajan, Hardy Kornfeld, Subash Babu

**Affiliations:** 1 National Institutes of Health—NIRT— International Center for Excellence in Research, Chennai, India; 2 Prof. M. Viswanathan Diabetes Research Center, Chennai, India; 3 National Institute for Research in Tuberculosis, Chennai, India; 4 University of Massachusetts Medical School, Worcester, MA USA; 5 LPD, NIAID, NIH, Bethesda, MD USA

**Keywords:** Predictive markers, Infection, Tuberculosis

## Abstract

Plasma cytokines are biomarkers of disease extent and mycobacterial burden in pulmonary tuberculosis (PTB). Whether chemokines can perform the same role in PTB is not known. We examined the plasma levels of chemokines in individuals with PTB, latent TB (LTB) or healthy controls (HC) and their association with disease severity and mycobacterial burdens in PTB. We also examined the chemokines in PTB individuals at the end of anti-tuberculous chemotherapy (ATT). PTB individuals exhibited significantly higher levels of CCL1, CCL3, CXCL1, CXCL2, CXCL9 and CXCL10 in comparison to LTB and/or HC individuals. PTB individuals with bilateral or cavitary disease displayed significantly elevated levels of CCL1, CCL3, CXCL1, CXCL10 and CXCL11 compared to those with unilateral or non-cavitary disease and also exhibited a significant positive relationship with bacterial burdens. In addition, PTB individuals with slower culture conversion displayed significantly elevated levels of CCL1, CCL3, CXCL1 and CXCL9 at the time of PTB diagnosis and prior to ATT. Finally, the chemokines were significantly reduced following successful ATT. Our data demonstrate that PTB is associated with elevated levels of chemokines, which are partially reversed followed chemotherapy. Our data demonstrate that chemokines are markers of disease severity, predicting increased bacterial burden and delayed culture conversion in PTB.

## Introduction

Tuberculosis (TB), a disease caused by *Mycobacterium tuberculosis* (Mtb), is the world’s leading cause of infectious disease mortality^1^. There are over 2 billion infected with Mtb worldwide and approximately 5–10% of infected individuals are thought to progress to active TB in their lifetime^1^. Infection with Mtb involves a spectrum of disease progression steps with latent infection and active disease being interspersed with incipient TB and subclinical TB^2^. Moreover, the clinical manifestations of TB disease are not uniform but might vary depending on the bacterial burden and disease severity^3^. Severe disease is thought to involve both the lungs (bilateral disease) and more of cavitary lesions, the latter of which is postulated to be associated with increased transmission^1^. Bacterial burden is reflected by the number of bacteria in the sputum as estimated by smear or culture. However, the immunological underpinnings of these disease manifestations remain incompletely understood.

Chemokines are felt to play a major role latent TB infection (LTB) as they appear to be critical in the formation and maintenance of quiescent granulomas^4^ and in the recruitment of cells from the periphery for positioning within the granuloma^5^. Establishment of the TB granuloma is controlled by the synchronized expression of various chemokines. However, chemokines, like cytokines, are a double-edged sword in infectious disease and can play a detrimental role in pathogenesis^6^. Thus, excessive levels of certain chemokines can drive the enhanced infiltration of neutrophils and monocytes to the site of infection, which might augment immune mediated pathology^6,7^. An optimal balance between protective and pathological immune responses at the site of infection in TB might be reflected by the levels of chemokines in the circulation^8^. Chemokines, categorized within four families (C, CC, CXC, CX3C), are essential in regulating inflammation, leukocyte recruitment and antimicrobial immunity^9,10^. The CC chemokines - CCL3, CCL4, CCL5 and CCL8 as well as the CXC chemokines - CXCL9, CXCL10 and CXCL11 are upregulated in *Mtb*-infected mice^6,11^. These chemokines play an important role in the recruitment of T cells and other cells to the lung during early infection^11^. Finally, defects in the production of certain chemokines have been associated with increased susceptibility to Mtb in animal models^5,6^.

Our previous work has also demonstrated that plasma cytokines are biomarkers of disease severity, mycobacterial burden and slower culture conversion in PTB^12^. To decipher the role of chemokines in TB infection and disease, we measured the levels of chemokines in PTB, LTB and HC individuals. Our goal was to correlate the levels of these chemokines with the extent and severity of disease, sputum bacterial burden, and time to culture conversion and to evaluate the longitudinal changes in chemokine levels before and after anti-TB treatment. Our data demonstrate that chemokines were indeed markers of disease severity, bacterial burden and time to culture conversion in PTB.

## Results

### Study demographics

All participants did not exhibit signs or symptoms of other lung disease. The individuals in the two groups were not significantly different in terms of age and gender and the baseline characteristics of the study participants are shown in Table 1. ATT was given to PTB individuals using the directly observed treatment, short course (DOTS) strategy. All PTB individuals were sputum culture negative at six months of treatment.

**Table 1:** Demographics of the study groups.

Study Demographics	PTB	LTB	HC
Number of subjects recruited	88	44	44
Gender (Male/Female)	61/27	30/14	28/16
Median Age (Range)	48 (25–70)	40 (25–67)	32 (23–55)
Smear Grade: 0/1+/2+/3+	0/32/27/29	—	—
Lung lesions (Bilateral/Unilateral)	56/32	—	—
Cavity (Cavity/No Cavity)	26/62		
Quantiferon TB Gold in Tube	—	Positive	Negative
Tuberculin Skin Test	—	Positive	Negative

### Plasma levels of chemokines are elevated in PTB

**Figure 1:**
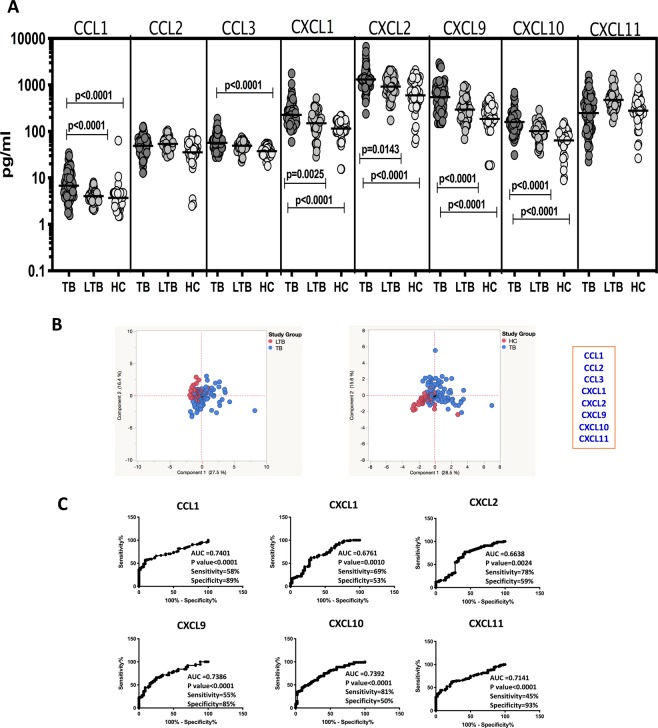
Elevated plasma levels of chemokines in PTB individuals (**A**) The plasma levels of chemokines were measured in PTB (n = 88), LTB (n = 44) and HC (n = 44) individuals. The data are represented as scatter plots with each circle representing a single individual. P values were calculated using the Kruskal-Wallis test with Dunn’s post hoc comparison. (**B**) PCA (Principle component analysis) plot computing normalized ELISA data from baseline plasma levels of cytokines in combination of two different experimental groups first PTB (Colored in blue) vs LTB (Colored in red) and second PTB (Colored in blue) vs HC (Colored in red). (**C**) ROC analysis to estimate the sensitivity, specificity and AUC was performed using chemokines to estimate the capacity of these factors to distinguish individuals with PTB vs. LTB.

To examine the systemic levels of chemokines in TB infection and disease, we measured the plasma levels of CCL1, CCL2, CCL3, CXCL1, CXCL2, CXCL9, CXCL10 and CXCL11 in PTB, LTB and HC individuals (Fig. 1). As shown in Fig. 1A, the systemic levels of CCL1 (Geometric Mean of 6.97 pg/ml in PTB versus 4.06 pg/ml in LTB and 3.69 pg/ml in HC), CXCL1 (GM of 226.1 pg/ml in PTB versus 150.3 pg/ml in LTB and 115.4 pg/ml in HC), CXCL2 (GM of 1309 pg/ml in PTB versus 921.3 pg/ml in LTB and 597.4 pg/ml in HC), CXCL9 (GM of 545.1 pg/ml in PTB versus 296.4 pg/ml in LTB and 186.1 pg/ml in HC) and CXCL10 (GM of 160.2 pg/ml in PTB versus 101.1 pg/ml in LTB and 63.8 pg/ml in HC) were significantly higher in PTB compared to both LTB and HC individuals, while the levels of CCL3 (GM of 56.7 pg/ml in PTB versus 49.3 pg/ml in LTB and 37.45 pg/ml in HC) was significantly higher in PTB compared to HC individuals. Next, PCA using the above set of chemokines clearly demonstrated the ability of these cytokines to discriminate PTB from LTB or HC individuals. Finally, ROC analysis of CCL1, CXCL1, CXCL2, CXCL9, CXCL10 and CXCL11 was performed in PTB vs LTB individuals (Fig. 1C). As shown in Fig. 1C, CCL1, CXCL9, CXCL10 and CXCL11 exhibited significant discriminatory power with moderate area under the curve (AUC) values and sensitivity and specificity in discriminating PTB from LTBI individuals. Thus, PTB individuals display significantly elevated systemic levels of chemokines.

### Plasma chemokines are markers of disease severity in PTB

**Figure 2:**
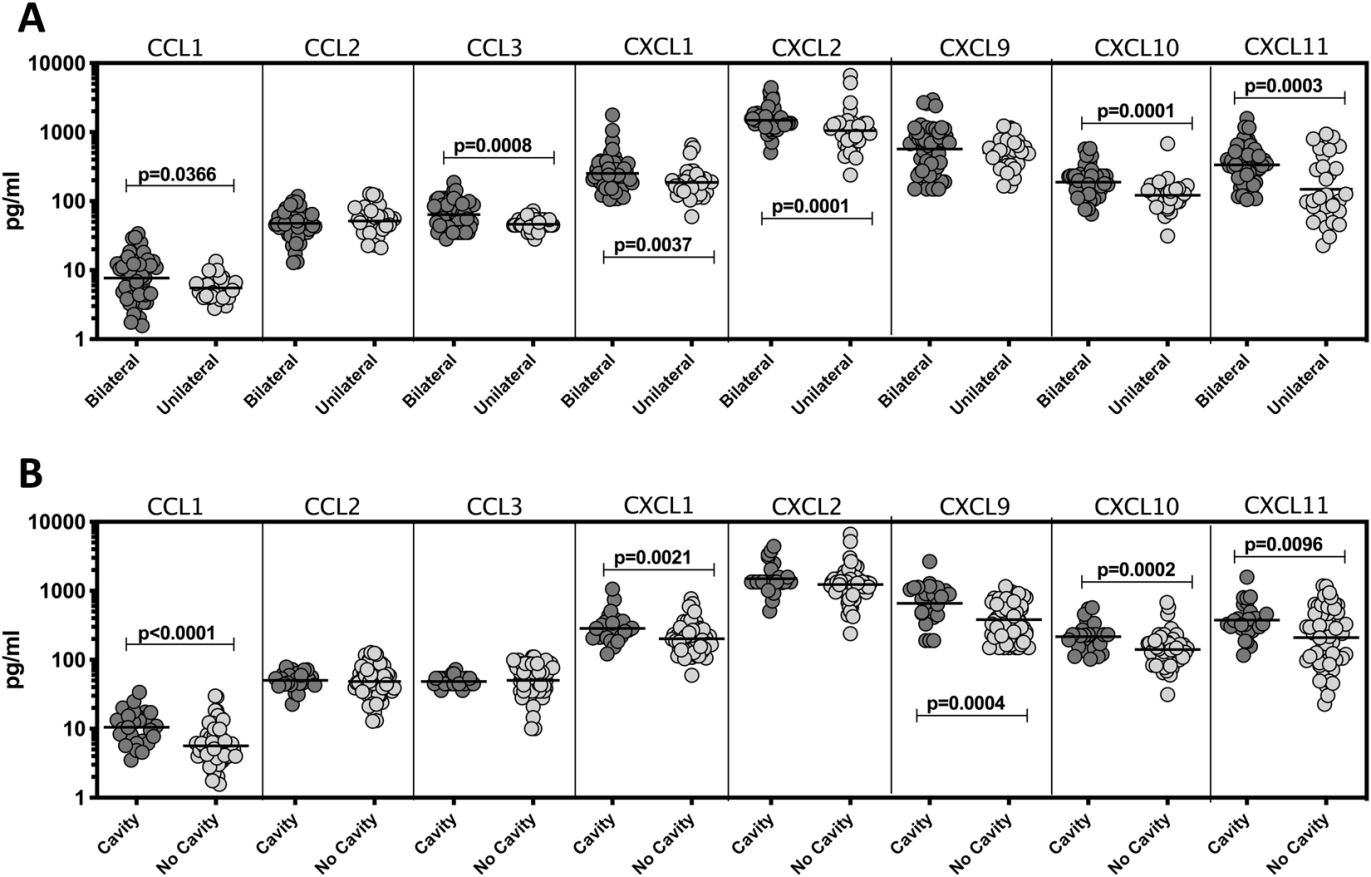
Plasma chemokines are associated with extent of disease, or disease severity in PTB individuals (**A**) The plasma levels of chemokines were measured in in PTB individuals with bilateral versus unilateral disease, reflecting the extent of disease. (**B**) The plasma levels of chemokines were measured in PTB individuals with cavitary versus non-cavitary disease, reflecting the disease severity. The data are represented as scatter plots with each circle representing a single individual. P values were calculated using the Mann-Whitney test with Holm’s correction for multiple comparisons.

To examine the association between the systemic levels of chemokines and disease severity and extent in PTB, we measured the plasma levels of CCL1, CCL2, CCL3, CXCL1, CXCL2, CXCL9, CXCL10 and CXCL11 in PTB individuals with unilateral versus bilateral disease and with cavitary versus non-cavitary disease (Fig. 2). As shown in Fig. 2A, the systemic levels of CCL1 (GM of 7.64 pg/ml in bilateral vs. 5.53 pg/ml in unilateral disease), CCL3 (GM of 63.98 pg/ml in bilateral vs. 46.08 pg/ml in unilateral disease), CXCL1 (GM of 251.5 pg/ml vs. 186.6 pg/ml), CXCL2 (GM of 1488 pg/ml vs. 1047 pg/ml), CXCL10 (GM of 187.8 pg/ml vs. 121.2 pg/ml) and CXCL11 (GM of 334.3 pg/ml vs. 148.3 pg/ml) were significantly increased in PTB individuals with bilateral disease compared to unilateral disease. Similarly, as shown in Fig. 2B, the systemic levels of CCL1 (GM of 10.51 pg/ml in cavitary disease vs. 5.65 pg/ml in non-cavitary disease), CCL3 (GM of 48.55 pg/ml vs. 50.22 pg/ml), CXCL1 (GM of 284.3 pg/ml vs. 202.2 pg/ml), CXCL9 (GM of 657.3 pg/ml vs. 381.8 pg/ml), CXCL10 (GM of 216.5 pg/ml vs. 141,1 pg/ml) and CXCL11 (GM of 375.4 pg/ml vs. 209.3 pg/ml) were significantly increased in PTB individuals with cavitary disease compared to those without cavitary disease. Thus, disease severity and extent in PTB is associated with increased plasma levels of chemokines.

### Plasma chemokines are markers of bacterial burdens and delayed sputum culture conversion in PTB

**Figure 3:**
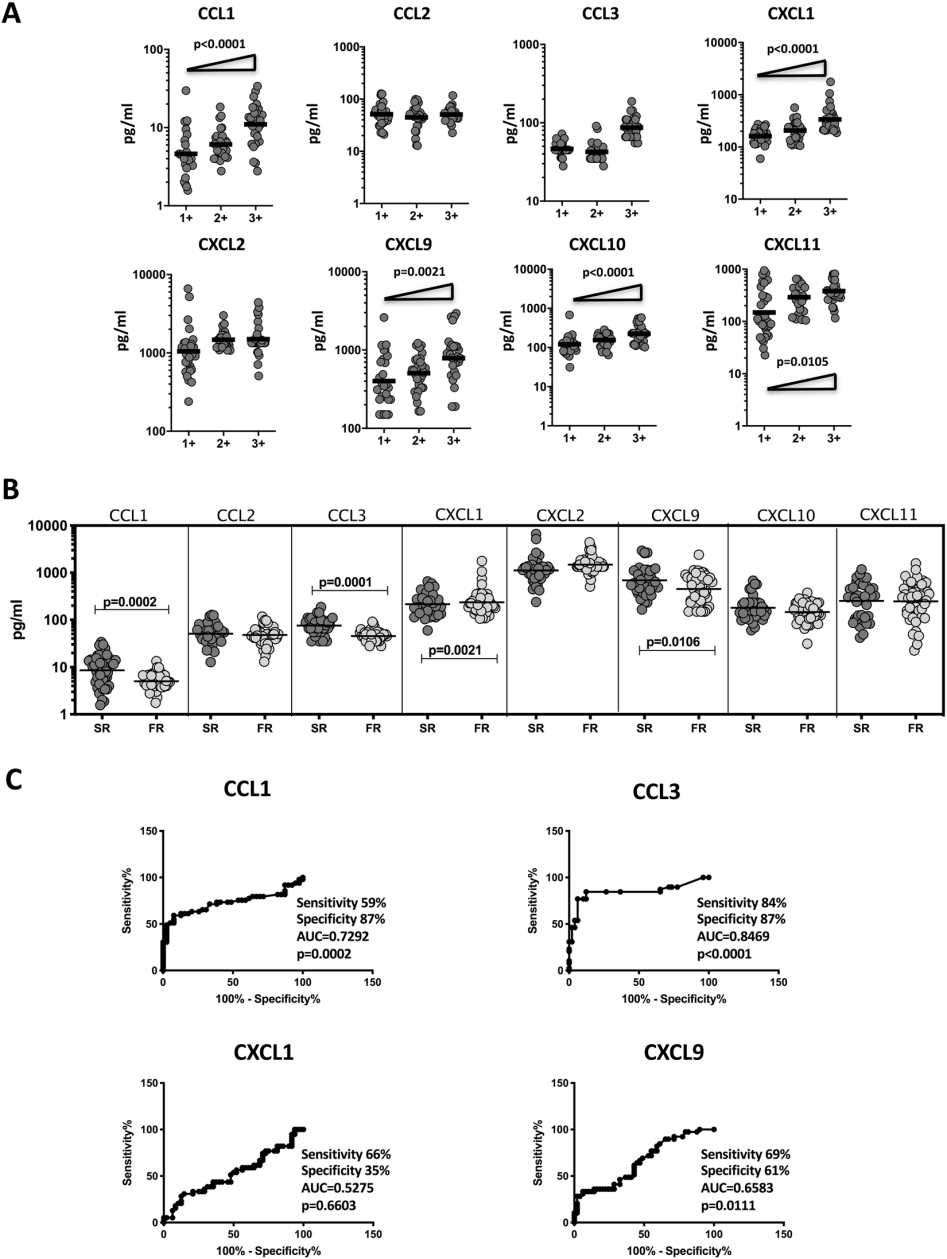
Plasma chemokines are associated with bacterial burdens in PTB individuals (**A**) The relationship between the plasma levels of chemokines and smear grades as estimated by sputum smears was examined in PTB individual. The data are represented as scatter plots with each circle representing a single individual. P values were calculated using the Linear trend post – test. (**B**) The plasma levels of chemokines at baseline were measured in PTB individuals, who were slow responders (Sputum positive during 2^nd^ month of ATT, SR) versus fast responders (Sputum negative during 2^nd^ month of ATT, FR). The data are represented as scatter plots with each circle representing a single individual. P values were calculated using the Mann-Whitney test with Holm’s correction for multiple comparisons. (**C**) ROC analysis to estimate the sensitivity, specificity and AUC was performed using chemokines to estimate the capacity of these factors to distinguish SR from FR.

To examine the association between the systemic levels of chemokines and bacterial burden in PTB, we performed a correlation of the plasma levels of CCL1, CCL2, CCL3, CXCL1, CXCL2, CXCL9, CXCL10 and CXCL11in PTB individuals with smear grade classified as 1+, 2+ and 3+ (Fig. 3). As shown in Fig. 3A, the systemic levels of CCL1, CCL2, CXCL1, CXCL9, CXCL10 and CXCL11 displayed a significant positive correlation with smear grades in PTB individuals, indicating a positive relationship of chemokines with bacterial burdens. To determine whether chemokines could serve as predictive biomarkers for delayed culture conversion in PTB, we measured the plasma levels of CCL1, CCL2, CCL3, CXCL1, CXCL2, CXCL9, CXCL10 and CXCL11 in PTB individuals at baseline (prior to commencement of treatment) and correlated to those who had positive cultures at 2 months (slow responders, SR) and those who had negative cultures at 2 months (fast responders, FR). As shown in Fig. 3B, the systemic levels of CCL1 (GM of 8.60 pg/ml in SR vs. 5.04 pg/ml in FR), CCL3 (GM of 75.34 pg/ml vs. 45.34 pg/ml), CXCL1 (GM of 215.2 pg/ml vs. 235.2 pg/ml) and CXCL9 (GM of 690 pg/ml vs. 451.8 pg/ml) were significantly higher in slow responders compared to fast responders. Finally, as shown in Fig. 3C, we also performed ROC analysis to determine the strength of plasma chemokines to distinguish SR from FR in the PTB individuals. While CCL1, CCL3 and CXCL9 all exhibited a significant area under the curve, only CCL3 exhibited high sensitivity and specificity in discriminating SR from FR. Combination of different chemokines did not provide additional predictive power over individual values. Thus, plasma chemokines are also markers of delayed culture conversion in PTB.

### Plasma chemokine levels are significantly diminished following ATT

**Figure 4:**
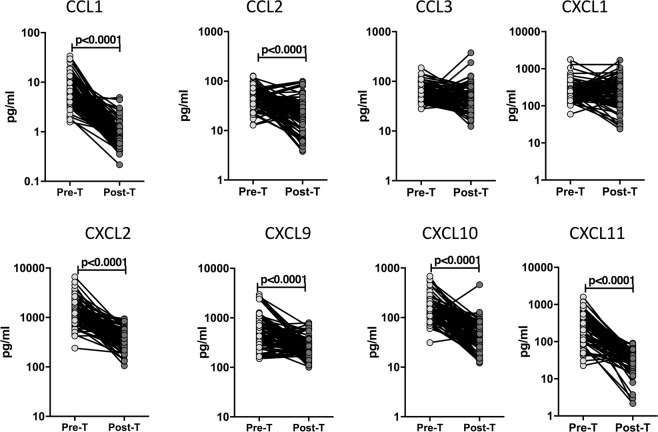
Diminished plasma levels of chemokines at the end of standard anti-tuberculosis therapy in PTB individuals. (**A**) The plasma levels of chemokines were measured in PTB at baseline (pre-T) and at 6 months of ATT (post-T). The data are presented as line graphs with each line representing a single individual. P values were calculated using the Wilcoxon signed rank test.

To examine whether the elevated levels of chemokines are directly linked with TB disease, we measured the levels of these chemokines in PTB individuals before and after a regimen of ATT (pre-T versus post-T). As shown in Fig. 4, at the end of ATT, the plasma levels of CCL1 (GM of 6.79 pg/ml pre-T vs. 0.88 pg/ml post-T), CCL2 (GM of 49.02 pg/ml vs. 19.37 pg/ml), CXCL2 (GM of 1309 pg/ml vs. 361.3 pg/ml), CXCL9 (GM of 545.1 pg/ml vs. 237.4 pg/ml), CXCL10 (GM of 160.2 pg/ml vs. 41.23 pg/ml) and CXCL11 (GM of 248.7 pg/ml vs. 28.71 pg/ml) were all significantly decreased compared to pre-treatment levels. Thus, chemokine levels are significantly decreased in PTB following successful chemotherapy.

## Discussion

Chemokines are major mediators of resistance in TB and these findings are mostly derived from animal models of infection^5^. However, these data also indicate that chemokines can serve as a double - edged sword with both the levels and timing of chemokine production serving as the balance of protection versus pathology^6^. Following infection, the interplay of pro-inflammatory and anti-inflammatory signals is important in the establishment and maintenance of the the tuberculosis granuloma^2,13^. A push towards a robust pro-inflammatory chemokine milieu can precipitate bacterial dissemination, with resultant changes in the granuloma architecture and damage of lung parenchyma^2,14^. These processes determine the activation of Tb disease and help promote transmission of infection. In our study, we demonstrate that heightened levels of chemokines are a major feature of PTB disease and serve as biomarkers of disease severity/extent and mycobacterial burden and can be modulated by chemotherapy.

Our data show that the CC chemokines – CCL1 and CCL3 as well as the CXC chemokines – CXCL1, CXCL2, CXCL9 and CXL10 are present at significantly elevated concentrations in the plasma of PTB individuals. It is possible that concentrations of chemokines at the site of infection has a more direct effect on host resistance to infection and disease. Our data also demonstrates the moderate sensitivity and specificity (by ROC analysis) offered by plasma chemokine measurements in distinguishing PTB from LTB individuals. In addition, our data reveals the lack of differences in the chemokine levels between latent TB infection and healthy controls. Future studies need to elucidate the role of these chemokine alterations in immune cell recruitment.

Biomarkers of TB disease severity and extent as well as blood markers of bacterial burdens are important tools in the progress towards elimination of TB^15^. Our data reveal that chemokines can serve as important biomarkers of disease severity (cavity or no cavity) and extent (unilateral or bilateral), since higher levels of CCL1, CCL3, CXCL1, CXCL10 and CXL11 are reflective of the disease severity and extent. In addition, CXCL2 for disease extent and CXCL9 for disease severity can also serve as plasma biomarkers. Our study also reveals that the same set of chemokines that serve as biomarkers of disease pathology can also serve as biomarkers of bacterial burdens and time to culture conversion. Thus, plasma levels of CCL1, CCL2, CXCL1, CXCL9, CXCL10 and CXCL11 are indicative of bacterial burdens with PTB individuals having the highest burdens exhibiting the highest levels of these chemokines. Moreover, PTB individuals who are culture positive at 2 months of ATT have significantly higher levels of CCL1, CCL3, CXCL1 and CXCL9 before commencement of ATT, indicating that baseline levels of these chemokines are markers of time to culture conversion and can be promising immunological predictors of culture conversion. Hence, measurement of plasma chemokines, especially CCL3, which could be done a baseline, might stratify patients who require intensified treatment e.g. high-dose rifampicin. This would particularly be the case if the chemokines outperformed baseline sputum smear grade for predicting outcomes, which needs to be tested in future studies. It should be noted that all PTB individuals were culture negative at the end of ATT and thus we could not ascertain the utility of these chemokines in prediction of treatment failure. Previous studies have shown that CXCL9 and CXCL10 are good biomarkers to distinguish PTB from LTB and that these markers significantly decrease following ATT^16,17^. Our study adds to this panel of host chemokines that can be used to monitor treatment responses since plasma levels of CCL1, CCL2, CXCL2, CXCL9, CXCL10 and CXCL11 are all significantly lowered following ATT.

Host biomarker studies have largely placed CXCL10 at the center of interest in the immunodiagnosis of TB^18,19^. Studies have shown that plasma levels of CXCL10 in active TB patients are significantly elevated after stimulation^20^, as well as exhibit greater sensitivity when discriminating between active TB and LTB, compared to using IFNγ alone^17,21^. The other promising chemokine studied thus far as a biomarker for TB is CXCL9^22–24^. Our study expands on this panel of chemokines and demonstrates that a larger panel of chemokines can serve as moderately good biomarkers of disease severity, bacterial burden and treatment monitoring. This includes CCL1, CCL3 and CXCL1. Our study has several positives including the large sample size, the use of bacteriologically proven (culture positive) PTB individuals with follow up and clearly delineated controls. However, our study only examines associations between chemokines and TB disease and cannot attribute any cause-effect relationship or delineate the mechanistic underpinnings of the role of chemokines in TB disease. Nevertheless, our data make a compelling argument for the continued evaluation of chemokines as an adjunct tool to evaluate TB disease in endemic populations and validate its utility for a point-of-care assay as a rule out or triage test for TB.

## Methods

### Ethics statement

This study was approved by the Ethics Committees of the Prof. M. Viswanathan Diabetes Research Center (ECR/51/INST/TN/2013/MVDRC/01) and NIRT (NIRT-INo:2014004). Informed written consent was obtained from all participants. All the methods were performed in accordance with institutional ethical committee guidelines. The study participants were recruited from the Effect of Diabetes in Tuberculosis Severity protocol conducted under the RePORT India consortium^25^. These participants were different from the participants in our previous study on cytokines in TB disease^12^, which was conducted at NIRT alone and this study was from a separate protocol.

### Study population

Plasma samples were collected by centrifuging the whole blood specimen at 2600 rpm for 10 mins from 88 individuals with active pulmonary TB (PTB), 44 individuals with latent TB (LTB) and 44 individuals with no TB infection or disease (HC), recruited in Chennai, India. Individuals with pulmonary TB were diagnosed by positive solid cultures in Lowenstein–Jensen medium and were classified as 1+ (10–100 colonies), 2+ (>100–200 colonies) and 3+ (>200 colonies). Chest radiographs on enrollment were graded by two blinded readers using a validated severity score based on the percent area of lung involved with TB disease and the presence or absence of cavities. Smear grades were used to determine bacterial burdens and classified as 1+ (10–99 AFB in 100 fields), 2+ (1–10 AFB in each field) and 3+ (more than 10 AFB in each field). Culture conversion (from positive to negative) at 2 months was defined as the cut-off for fast responders (FR, who culture converted) versus slow responders (SR, who were still culture positive). At the time of enrollment, all active TB cases had no record of prior TB disease or anti-TB treatment (ATT). Tuberculin skin test was performed using two tuberculin units of Tuberculin Purified Protein Derivative RT 23 Serum Statens Institute. A positive skin test was defined as an induration of at least 12-mm diameter based on the previously determined cutoff norms for South India and Quantiferon TB-Gold in Tube ELISA positivity, absence of chest radiograph abnormalities or pulmonary symptoms and negative sputum smears. HC individuals were asymptomatic with normal chest X-rays, negative TST (indurations <5 mm in diameter) and Quantiferon results. Study enrolled participants were BCG vaccinated, HIV negative, non-diabetic and had normal body mass index (BMI, between 18.5 and 24.9 kg/m2).

### ELISA

Circulating levels of chemokines were measured using Bio-Plex multiplex assay system (Bio-Rad, Hercules, CA). Luminex Human Chemokines Magnetic Assay kit (R & D systems) was used to measure the chemokine levels. The lowest detection limits were as follows: CCL1, 1.57 pg/mL; CCL2, 31.8 pg/mL; CCL3, 90.9 pg/mL; CXCL1, 49.2 pg/mL; CXCL2, 49.2 pg/mL; CXCL9, 600.6 pg/mL CXCL10, 2.88 pg/mL and CXCL11, 21.6 pg/mL.

### Statistical analysis

Geometric means (GM) were used for measurements of central tendency. Statistically significant differences between the three groups were analyzed using the Kruskal-Wallis test with Dunn’s multiple comparisons. Principal component analysis (PCA) was done using statistical software JMP 13.0 (SAS, Cary, NC, USA). Receiver operator characteristics (ROC) curves were designed to test the power of each candidate chemokine to distinguish LTB from PTB and SR from FR in PTB. Mann-Whitney test was used to compare chemokine concentrations in PTB individuals with unilateral or bilateral lung lesions or cavitary or non-cavitary disease and slow responders or fast responders with Holm’s correction for multiple comparisons. Linear trend post-test was used to compare chemokine concentrations with smear grades (reflecting bacterial burdens). Wilcoxon signed rank test was used to compare chemokine concentrations before and after ATT. Analyses were performed using Graph-Pad PRISM Version 7.0.

## Data Availability

All data are available in the manuscript and figures.
